# The emerging roles of N6-methyladenosine RNA methylation in human cancers

**DOI:** 10.1186/s40364-020-00203-6

**Published:** 2020-06-29

**Authors:** Huafei Shen, Yifen Lan, Yanchun Zhao, Yuanfei Shi, Jie Jin, Wanzhuo Xie

**Affiliations:** 1 Department of Hematology, the First Affiliated Hospital of Medical School of Zhejiang University, No. 79 Qingchun Road, Hangzhou, 310003 Zhejiang China; 2 Department of Hematology, Lishui People’s Hospital, No. 15 Dazhong Road, Lishui, 323000 Zhejiang China

**Keywords:** N6-methyladenosine, RNA methylation, Tumorigenesis, Cancer, Epigenetics, Therapy

## Abstract

N6-methyladenosine (m^6^A) is the most abundant form of mRNA modification in eukaryotes. It affects various aspects of RNA metabolism, including nuclear export, translation, decay and alternative splicing. In addition, m^6^A also participates in a great number of human physiological processes, ranging from spermatogenesis modulation, response to heat shock, the control of T cell homeostasis to stem cell proliferation and differentiation. The dynamic equilibrium of m^6^A level is regulated by m^6^A methyltransferases (“writers”), m^6^A demethylases (“erasers”) as well as m^6^A-binding proteins (“readers”). Once the balance is broken, numerous diseases will knock on the door. Recently, increasing studies reveal that m^6^A methylation exerts a profound impact on tumorigenesis and tumor progression. Therefore, in this review, we summarize the functions of m^6^A modification and its emerging roles in human cancers, and discuss the potential of m^6^A regulators as biomarkers or therapeutic targets.

## Background

Cancer is a major public health problem worldwide and one of the most important causes of death. According to data released by the American Cancer Society in 2020, there will be more than 1.8 million new cancer cases and over 600 thousand people will die of cancer in the United States [1]. Therefore, it is of great importance to further explore the pathogenesis of tumors and improve the treatment effect.

With rapid development of detecting methods, especially high-throughput sequencing [2], increasing evidence shows that N6-methyladenosine (m^6^A) is the most abundant type of mRNA modification in eukaryotes, accounting for about 0.1–0.4% of adenosine nucleotides [3, 4]. The consensus motif of m^6^A is considered as RR(m^6^A) CH sequences (R = G/A/U, R = G/A, H=U/A/C) [5, 6]. Furthermore, it mainly gathers in 5′ or 3′ untranslated regions (UTRs), near stop codons and within internal long exons [2, 7], revealing that it may be involved in switching genes on/off and/or regulating the binding of proteins linked with the downstream biological functions. As a matter of fact, it is true that m^6^A, as a key modification, plays a role of Moirai in the fate of mRNAs, including their nuclear export, translation, decay and alternative splicing [2, 8–13]. Several studies have shown that m^6^A participates in physiological processes including spermatogenesis modulation, response to heat shock, control of T cell homeostasis and stem self-renewal and differentiation [14–17].

**Fig. 1:**
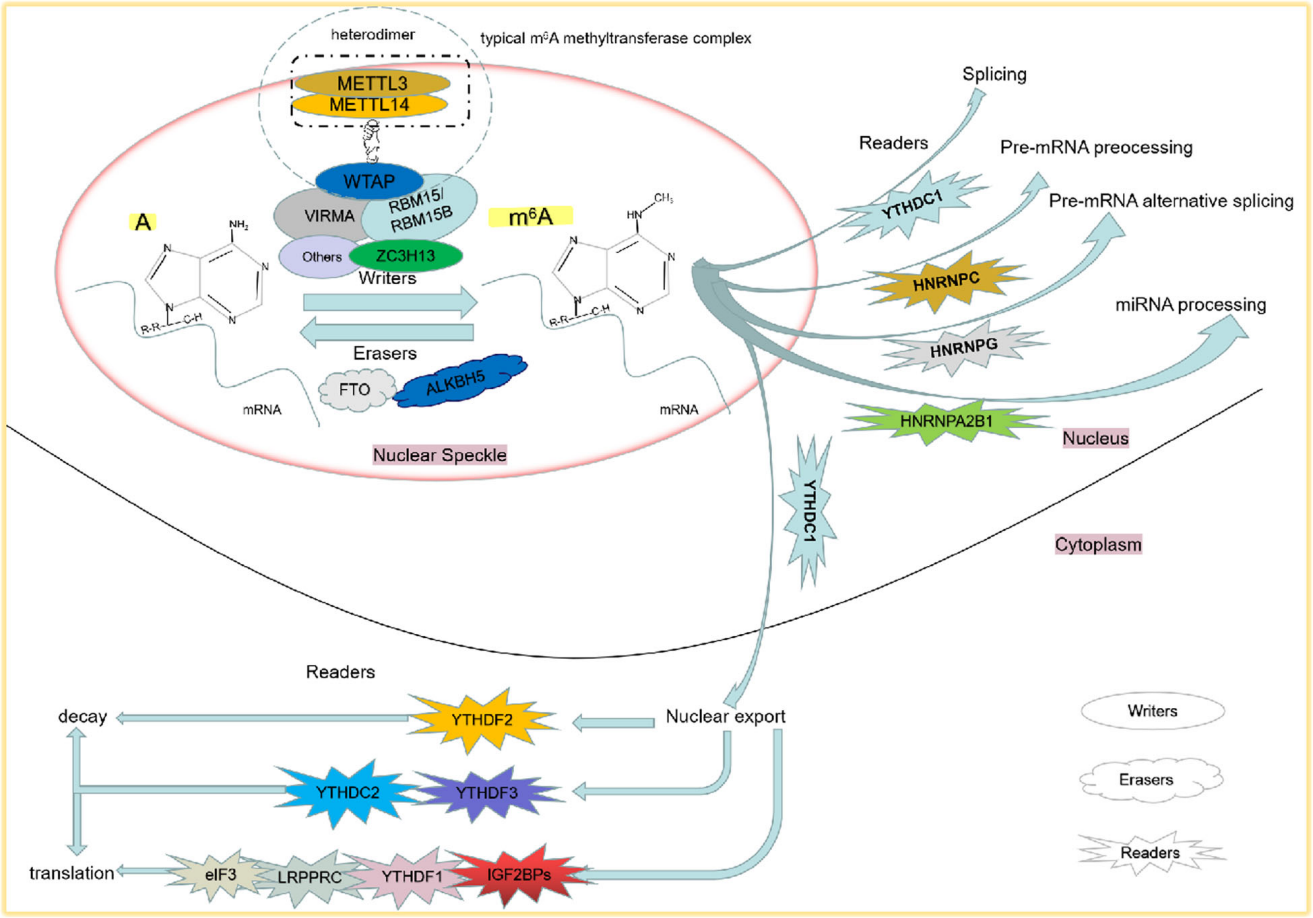
The progress of m^6^A RNAs methylation. The integrity of m^6^A RNAs methylation modification is maintained by writers, erasers readers interact with RRACH, the specials sequence, to complete the installation of m^6^A, and the removal is the duty of erasers, Readers primarily decide the fate of m^6^A RNAs by recognizing and binding with them and then helping them in metabolism

The constant discovery of enzymes regulating m^6^A modification enables us to have a better understanding of the whole process of m^6^A RNA methylation [9, 18–20]. The modification process of m^6^A is dynamic and reversible, which is mainly regulated by three kinds of enzymes: methyltransferases (“writers”), demethylases (“erasers”) as well as m^6^A-binding proteins (“readers”). As their names suggest, they add, remove and recognize m^6^A respectively. Under the joint efforts of these enzymes, the level of m^6^A maintains a dynamic equilibrium (Fig. 1).

Recently, a lot of studies have found that the aberrant expression of m^6^A regulatory enzymes is associated with human diseases, particularly cancers. In this review, we elaborate on the specific functions of m^6^A methylation, and summarize its emerging roles in tumorigenesis. Moreover, we also discuss the potential of m^6^A regulators as biomarkers or therapeutic targets in human cancers.

### The process and molecular functions of m^6^A RNA methylation

#### RNA m^6^A methylation is accomplished by methyltransferases

Before we elaborate on the m^6^A RNA methylation process, let’s first recognize some important methyltransferases (“writers”). First, methyltransferase-like 3 (METTL3) is regarded as the major catalytic enzyme of m^6^A methylation, which binds to S-adenosylmethionine (SAM) and then activates the methylation activity [18, 21, 22]. Second, methyltransferase-like 14 (METTL14), another active methyltransferase, is considered to be closely related to METTL3 [23]. With the continuous development of detection methods and increasing attention to m^6^A, more and more methyltransferases have been found, including Wilms tumor 1-associated protein (WTAP), virilizer like m^6^A methyltransferase associated protein (VIRMA, also known as KIAA1429), RNA binding motif protein 15 (RBM15) and its paralogue RBM15B, zinc finger CCCH-type containing 13 (ZC3H13), Cb1 proto-oncogene like 1 (CBLL1) and methyltransferase-like 16 (METTL16) [18, 20, 24–27].

m^6^A mRNA methylation is an intricate process catalyzed by a core methyltransferase complex. METTL3 is combined with METTL4 at 1:1 to form a heterodimer. And METTL14 promotes the methylation of METTL3. What’s more, WTAP, a cofactor of the heterodimer, is one of the members of the core methyltransferase complex. WTAP locates the heterodimer formed by METTL3 and METTL4 to nuclear speckle where m^6^A RNA methylation is completed [18, 20, 22, 28]. At this point, the draft of methylation process has been finished. Moreover, due to the increasing attention to m^6^A, more writers’ functions are gradually discovered. Besides the METTL3/METTL14/WTAP complex, other enzymes mainly play an auxiliary role. The absence of VIRMA contributes to the prolongation of numerous mRNA 3’UTR, and there is obvious overlap in the target transcripts, which suggests that it can promote the recruitment of METTL3/METTL14/WTAP complex to 3’UTR and stop codons [27]. RBM15 and its paralogue RBM15B are RNA structure identifiers, contributing to the occurrence in specific sequences of m^6^A mRNA methylation by interacting with WTAP via ZC3H13 [29, 30]. What’s more, a study suggests that lncRNA XIST is one of the targeted RNAs of RBM15/RBM15B. Thus, it is not difficult to imagine that they play indispensable roles in the X-inactivation and gene silencing [29]. And ZC3H13, located in nucleus, further promotes the correct location of WTAP, VIRMA and CBLL1 [30]. METTL16 has been proved to be an m^6^A methyltransferase recently, which is mainly responsible for the methylation of U6 spliceosome RNA [26, 31, 32]. In addition, many methyltransferases are still undiscovered and the functions of those identified methyltransferases remain unclear.

#### RNA m^6^A methylation is removed by demethylases

m^6^A modification was not regarded to be dynamic and reversible until the discovery of fat mass and obesity-associated protein (FTO) [19]. Then came the identification of AlkB homologue 5 (ALKBH5). Both FTO and ALKBH5 function as the well-known demethylases, abrogating the m^6^A methylation of mRNAs in the nuclear [8, 9, 33]. Interestingly, these two enzymes both belong to the AlkB family of Fe (II)/α-ketoglutarate-dependent dioxygenases. However, they target different mRNAs [9, 34]. A recent study finds that their selection of mRNAs largely relies on the structure and conformation of mRNAs caused by m^6^A accumulation rather than the consensus m^6^A sequence motif [35]. In addition, other demethylases are gradually discovered, such as AlkB homologue 3 (ALKBH3) [36]. Different from FTO and ALKBH5, ALKBH3 prefers tRNA to mRNA and rRNA [37].

#### RNA m^6^A methylation is recognized by m^6^A-binding proteins

m^6^A-binding proteins serve as the performers of m^6^A methylation, affecting RNA metabolism. YT521-B homology (YTH) domain family, the most-studied readers, recognize m^6^A modification by forming a hydrophobic aromatic cage composed of β strands and α helices [38–40]. The YTH domain family is mainly comprised of YTH domain family protein 1–3 (YTHDF1–3) and YTH domain containing protein 1–2 (YTHDC1–2). YTHDF1 improves the translation efficiency of targeted mRNAs via cooperating with translation initiation mechanism [38]. On the contrary, YTHDF2, the first identified m^6^A-binding protein, stimulates the decay of targeted mRNAs by recruiting the CCR4-NOT deadenylase complex [41]. Surprisingly, YTHDF3 not only enhances the translation of methylated mRNAs in synergism with YTHDF1, but also promotes the decay effect of YTHDF2 [11, 42]. YTHDC1, located in nucleolus, is linked to alternative splicing, nuclear export and X chromosome genes transcriptional silencing [43, 44]. Intriguingly, YTHDC2 can also promote the decay and translation of targeted mRNAs, similar to the dual function of YTHDF3 [45–47].

Notably, some members of heterogeneous nuclear ribonucleoproteins (HNRNP) family also possess the capacity to recognize m^6^A RNA methylation. Heterogeneous nuclear ribonucleoproteins A2/B1 (HNRNPA2B1), a nuclear m6A-binding protein, is involved in primary miRNA processing and maturation through the cooperation with DGCR8 protein [48]. Moreover, there is a phenomenon termed as “m^6^A-switch” that the mRNA abundance and splicing are impacted by heterogeneous nuclear ribonucleoproteins C (HNRNPC) and heterogeneous nuclear ribonucleoproteins G (HNRNPG) in an m^6^A -dependent manner and that m^6^A strengthens the binding of transcripts to HNRNPC and HNRNPG in turn by affecting the secondary structure of RNA [49, 50]. Besides, insulin-like growth factor 2 mRNA-binding proteins (IGF2BPs, including IGF2BP1/2/3) have been proved to have the role of “readers” like YTH domain family protein 1–3 and HNRNP family. In function, IGF2BPs can inhibit decay, strengthen storage and accelerate translation, which contributes to facilitating transcript [51].

What’s more, some non-classical readers have been discovered. Eukaryotic initiation factor 3 (eIF3) plays a role in promoting the cap-independent translation [52]. To our surprise, apart from the methyltransferase activity, METTL3 can promote translation of m^6^A-containing transcripts as a reader in certain cell types [53]. Additionally, there are some studies demonstrating that leucine rich pentatricopeptide repeat containing (LRPPRC) and fragile X mental retardation protein (FMRP) are also able to recognize the m^6^A modifications [54–56].

In conclusion, the integrity of m^6^A RNAs methylation modification is maintained by writers, erasers and readers (Fig. 1). Its dynamic balance enables the body to maintain normal physiological function. Correspondingly, the aberrant expression of m^6^A regulatory enzymes leads to human diseases, which provides a novel idea to study the mechanism of tumorigenesis.

### The role of m^6^A RNA methylation in cancers

It has been proved in lots of studies that the aberrant expression of m^6^A is associated with various kinds of tumors, ranging from solid cancers to hematological malignancies. However, the role of m^6^A in cancers is more like a double-edged sword, extending beyond carcinogenesis to a suppressor. Herein, we briefly review the function of m^6^A in different cancers in recent studies.

### Nervous system

#### Glioblastoma (GBM)

GBM is one of the most common and destructive brain tumors, with a median survival of only 1 year [57]. Recently, there are several studies demonstrating that GBM is closely related to overexpression of METTL3, which can promote the stemness of glioma stem cells (GSCs) [58, 59]. The excessive proliferation of GSCs which can stimulate the tumor growth and invasion, is the chief culprit of resistance to chemoradiotherapy as well [60]. Mechanistically, on the one hand, METTL3 reinforces the structure of SRY-related high-mobility-group (HMG)-box protein-2 (SOX2) RNA by recruiting of human antigen R (HuR), an RNA-binding protein, in an m^6^A-dependent manner. It contributes to the maintenance of GSCs stemness [58]. Knockdown of METTL3 significantly improves the response of GBM patients to radiotherapy and chemotherapy and is associated with more favorable outcomes [58]. On the other hand, the level of m^6^A serine-and arginine-rich splicing factors (SRSFs) can be increased by METTL3, which raises the nonsense-mediated mRNA decay (NMD) of SRSFs by YTHDC1. And the decrease of SRSFs influences the alternative splicing isoform switches, ultimately resulting in the hyperactivity of GSCs [59]. However, there are different voices about the role of METTL3 in GBM [60, 61]. These studies suggest that the downregulation of METTL3/METTL14 strengthens the stemness of GSCs by decreasing the level of m^6^A on ADAM19, which leads to the overexpression of ADAM in GSCs.

Apart from writers, the aberrant expression of erasers also has supportive impacts on GBM. According to the current researches, both FTO and ALKBH5 are overexpressed in GBM. There may be several explanations for this phenomenon. First, up-regulation of FTO and/or ALKBH5 leads to a decrease in m^6^A ADAM level through demethylation. As mentioned in the previous paragraph, it causes to an increase in the expression of ADAM in GSCs, which in turn significantly enhances the self-renewal ability of GSCs and hinders differentiation [58]. Second, one study has shown that overexpression of ALKBH5 can lead to down-regulation of m^6^A Forkhead box protein M1 (FOXM1) by decreasing FOXM1 nascent transcripts. Accordingly, the number of HuR recruited to FOXM1 pre-mRNA increases, promoting its stability to facilitate its expression and finally contributing to the GSCs proliferation [33, 62].

The role of METTL3 in GBM is still controversial, which encourages more researchers to dig deeper for the possibility of future treatment. Luckily, some inhibitors of FTO like meclofenamic acid 2 (MA2) have shown the efficiency in GBM treatment [60, 63]. Also, the knockdown of ALKBH5 also provides the potential possibility of treatment, worthy of further exploration.

### Respiratory system

#### Lung cancer (LC)

Lung cancer is one of the cancers with the highest morbidity and terrible mortality worldwide, whose 5-year survival rate is less than 20% [64]. Lung adenocarcinoma (LUAD), the most common histological manifestation of LC, is closely related to the abnormal of m^6^A [65–69]. One of these studies shows that METTL3, RBM15, VIRMA, YTHDF1, YTHDF2, HNRNPC and HNRNPA2B1 are significantly up-regulated in LUAD. Surprisingly, the overexpression of METTL3, YTHDF1 and YTHDF2 are linked to better overall survival and recurrence-free survival [66]. Based on these, another study reveals that the expressions of METTL14, ZC3H13, WTAP and FTO are down-regulated in LUAD [68]. On the contrary, some studies have shown that FTO is overexpressed as a carcinogenic factor in lung cancer [67]. Collectively, ALKBH5 also has been proved to be elevated in the LUAD [69].

**Fig. 2:**
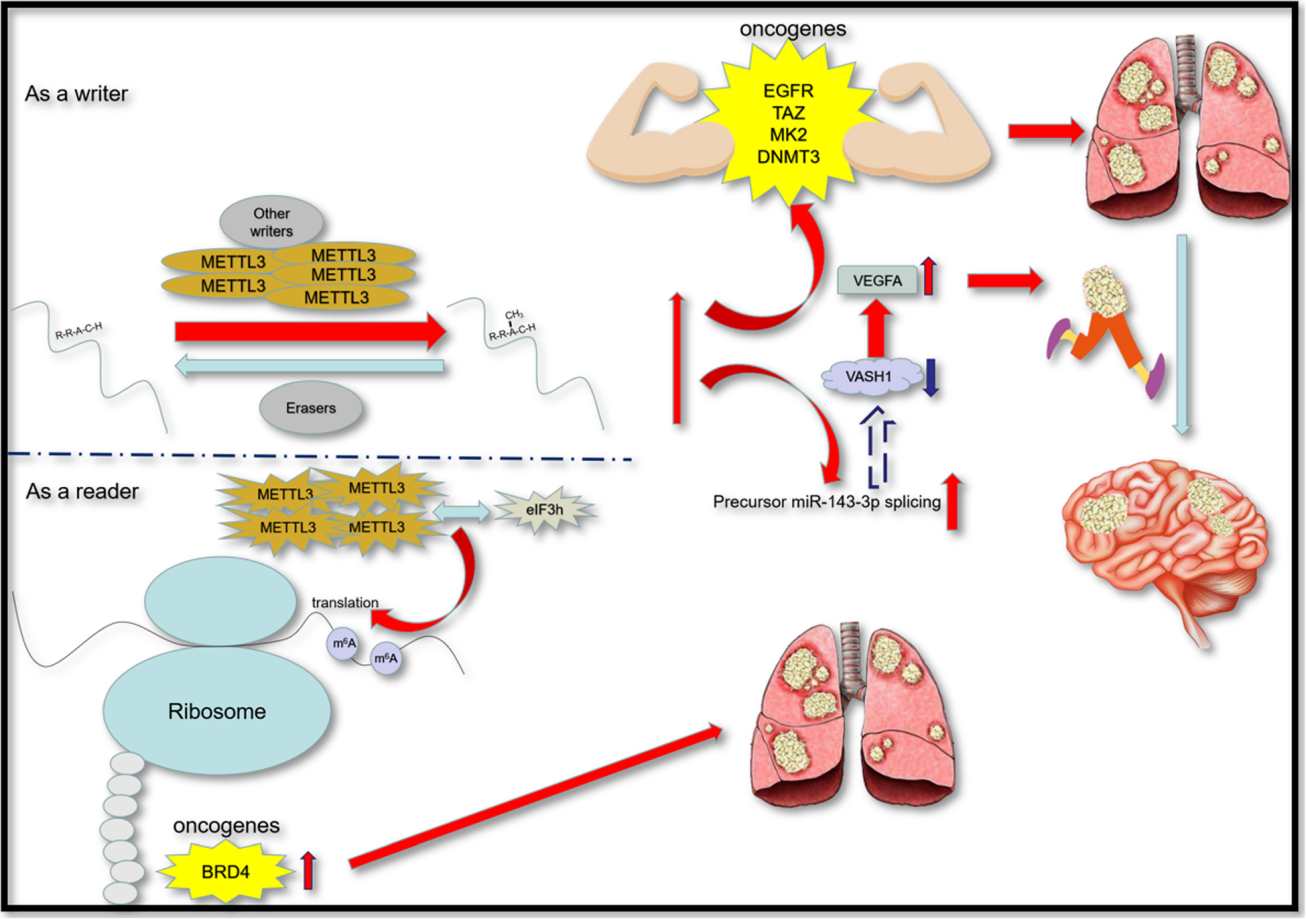
The mechanism of METTL3 in LC as on oncogenic role. The up-regulated METTL3 can promote the expression of some oncogenes like EGFR, TAZ, MK2, DNMT3 in am m^6^A-dependent manner and finally leads to LC, And it can also inhibit VASH1 by stimulating the spicing of precursor miR-143-3p, which is responsible for the brain metasis. As a writer, METTL3 can facilitate the translation of some oncogenes such a BRD4 and ultimately contribute to the LC/

Mechanistically, compared with other m^6^A regulators, the role of METTL3 has been better-studied (Fig. 2). As a writer, METTL3 targets oncogenes like EGFR, TAZ, MK2 and DNMT3, and enhances the expression of them to nourish the cancer cells with better survival, growth and invasion [53]. Moreover, METTL3 can up-regulate the level of miR-143-3p by promoting splicing of its precursor. MiR-143-3p can inhibit the expression of vasohibin-1 (VASH1) by interacting with three binding sites of 3’UTR of it. And VASH1 can facilitate the cell movement by increasing the ubiquitylation of vascular endothelial growth factor (VEGFA), which contributes to the brain metastasis of LC [70]. What’s more, METTL3 also serves as a reader to facilitate the progression of LC by interacting with the subunit h of eIF3 (eIF3h) directly in polyribosomes. They together promote the overexpression of bromodomain-containing protein 4 (BRD4), another criminal of the tumorigenesis [71]. As for FTO, despite the controversy, it is clear that it reduces the m^6^A modification on its targeted mRNAs including laminin γ2 (LAMC2), thrombospondin 1 (TSP1), nerve growth factor inducible (VGF), integrin alpha11 (ITGα11), and proprotein convertase subtilisin/kexin type 9 (PCSK9). Finally, it promotes to the proliferation, migration, and invasion ability of cancer cells [67]. As for ALKBH5, just like mentioned in the GBM, the overexpression of this eraser results in the increase translation efficiency of FOXM1 mRNA by decreasing the level of m^6^A in FOXM1, which promotes the growth of not only GSCs but also LUAD cells [33, 62, 69].

Lung squamous cell carcinoma (LUSC), another subtype of LC, accounts for about 1/3 of LC and leads to 400,000 deaths every year worldwide [72, 73]. And a study demonstrates that METTL3, ALKBH5, HNRNPC and VIRMA are related to the poor prognosis of LUSC, especially in the low-risk patients [74]. However, the mechanism is not clear yet and calls for more researches.

### Digestive system

#### Hepatocellular carcinoma (HCC)

When it comes to the role of m^6^A in cancers, the topic of HCC is inevitable for its heat. HCC, the main type of liver cancer, has high morbidity and mortality [75]. METTL3, WTAP, VIRMA, FTO, YTHDF1 and IGF2BPs have turned out to be up-regulated in HCC as oncogenic roles. However, the roles of METTL14 and YTHDF2 are still controversial.

Mechanistically speaking, METTL3 targets the suppressor of cytokine signaling 2 (SOCS2), a cancer suppressor, and increases the m^6^A level of it. The SOCS2 modified by m^6^A methylation can be recognized by YTHDF2 and further degraded, which eventually leads to the occurrence of HCC [76]. What’s more, another study notes that by interacting with YTHDF1, METTL3 can also function as a writer to facilitate the translation of Snail. And Snail is a crucial transcription factor in epithelial–mesenchymal transition (EMT), which is associated with HCC [77]. Besides, a study demonstrates that LINC00958, a lipogenesis-related Long non-coding RNAs (lncRNAs), is overexpressed in HCC due to the m^6^A methylation mediated by METTL3, which promotes the hepatoma-derived growth factor (HDGF) expression by inhibiting miR-3619-5p [78]. In HCC, WTAP has not received much attention until last year when a study pointed out the oncogenic role and mechanism of WTAP in HCC. The study notes that WTAP can promote the m^6^A methylation of ETS proto-oncogene 1 (ETS1), a suppressor of HCC progression. With the help of HuR, WTAP inhibits the translation of ETS1. Furthermore, WTAP-ETS1 axis can regulate the G2/M phase of HCC cells in a p21/p27-dependent pattern [79]. Similarly, VIRMA can induce the m^6^A methylation of ID2, which leads to the decrease of ID2 proteins, and eventually promotes the migration and invasion of HCC [80]. Moreover, VIRMA can also facilitate the m^6^A methylation of GATA3, conducing to the separation of HuR, a RNA stabilizer. Later, GATA3 ends up with decay [81].

As a representative of erasers, FTO mediates the demethylation of PKM2 and promotes its translation, which accelerates the progression of HCC [82]. IGF2BPs are also been proved to be related to the HCC. They can recognize the m^6^A methylated oncogenes like MYC and enhance its expression by strengthening the stability [51].

Finally, let’s talk about these two controversial m^6^A regulators with potential dual roles in HCC. It is generally believed that the level of the METTL14 is down-regulated in HCC and has to be responsible for the metastatic and relapse, which leads to the decrease of m^6^A methylation in miR126. And miR126 is a tumor suppressor and needs to be matured by DGCR8 in the form of m^6^A methylation [83]. However, Chen et al. demonstrate that METTL14 is unchanged in their transcriptome sequencing analysis [76]. In addition to the accelerated degradation of SOC2 mediated by the overexpression of YTHDF2 mentioned above, some researchers argue that the expression of YTHDF2 is down-regulated due to the hypoxia, which reduces the degradation of m^6^A methylated interleukin-11 (IL11) and serpin family E member 2 (SERPINE2). The increases of IL11 and SERPINE2 induce the inflammation and vascular remodeling, and eventually devote to the HCC [84].

From the perspective of therapy, the METTL3/SOCS2/YTHDF2 axis can be inhibited by miRNA145 through regulating the level of YTHDF [85]. In terms of another mechanism, PT2385, a hypoxia induced factor 2a (HIF-2a) inhibitor, can improve the hypoxic environment to rescue the down-regulated YTHDF2 and decrease the expression of IL11 and SERPINE2 [84]. In addition, there is a PLGA-based nanoplatform which is loaded with si-LINC00958. The new drug is gradually entering the market for the treatment of HCC [78].

#### Gastric cancer (GC)

GC, one of the most common malignancies worldwide, has a high morbidity, especially in middle-aged and elderly men [75]. METTL3 acts as an oncogenic role in GC with several mechanisms. First of all, METTL3 can trigger m^6^A methylation in MYC mRNA and promote its translation [86]. Secondly, METTL3 inhibits the apoptosis of GC cells by facilitating the expression of Bcl2 which can competitively combine with Bax, an apoptosis inducing factor [10]. Third, METTL3 can also cause the progression of GC by over-activating the AKT pathway, a crucial regulator involved in many key progressions like promoting cell proliferation and survival [87]. Fourth, METTL3 also targets SEC62 mRNA and triggers m^6^A methylation. The m^6^A-modified SEC62 mRNA can be recognize by IG2FBP1, which promotes its translation [88]. Fifth, analogously, METTLE3 facilitates the m^6^A methylation of HDFG mRNA to help it integrate with IGF2BP3, and thus increases the translation [89]. Besides, ALKBH5 can down-regulate the level of m^6^A nuclear paraspeckle assembly transcript 1 (NEAT1) mRNA by demethylation, which increases its expression. NEAT1 can be used as a cofactor to affect the expression of enhancer of zeste homolog 2 (EZH2) and it can also promote tumor invasion and metastasis [90].

#### Colorectal cancer (CRC)

Although the treatments are becoming more and more effective, the 5-year survival rate of CRC is only 64.9% [91]. Several studies show that the m^6^A regulators such as METTL3, METTL14, YTHDF1, YTHDF2 and YTHDF3 are involved in the tumorigenesis of CRC. There are two views on the role of METTL3 in CRC. One of them regards METTL3 as an oncogene by METTL3/SOX2/stem cells axis in an m^6^A-IGF2BP2-dependent mechanism [92, 93]. Another considers METTL3 as a suppressor by targeting at p38/ERK signaling pathways [94]. As for another key methyltransferase, METTL14 is regarded as an oncogene in CRC. In the mechanism, it can up-regulate the m^6^A level of lncRNA XIST, which is recognized by YTHDF2. And then YTHDF2 mediates the degradation of XIST, which suppresses the proliferation and metastasis of CRC [95]. Additionally, YTHDF1 is able to improve the stem cell-like activity in CRC by activating the Wnt/beta-catenin pathway [96]. Moreover, a recent study finds that YTHDF3 plays a key role in YES-associated protein (YAP) signaling by accelerating the decay of m^6^A-modified lncRNA GAS5, which promotes the progression of CRC [97].

#### Pancreatic cancer

It is well known that the fatality rate of pancreatic cancer is staggering and its treatment is very limited. Therefore, it is very important to study its pathogenesis. The overexpression of FTO, IGF2BP2 and YTHDF2 are related to the pancreatic cancer. A study notes that knockdown of FTO inhibits the proliferation of cancer cells and induces them to apoptosis, suggesting that FTO plays an oncogenic role. Mechanistically, FTO contributes to the proliferation of pancreatic cancer cells by decreasing the m^6^A of MYC and basic Helix-Loop-Helix transcription factor (bHLH-TF) and then promoting their translation [98]. And IGF2BP2, as a reader, binds to m^6^A-modified DANCR which devotes to the maintenance of stemness of cancer cells. In this way, the translation of DANCR is enhanced [99]. Moreover, the expression of YTHDF2 is up-regulated in pancreatic cancer. YTHDF2 has an influence on EMT by YAP signaling, which finally leads to the pancreatic cancer. Surprisingly, YTHDF2 also has the ability to inhibit the migration of cancer cells in pancreatic cancer [100].

### Urinary system

#### Clear cell renal cell carcinoma (ccRCC)

ccRCC is the most common form of kidney cancer, accounting for about 70% of adult kidney cancers [101]. A study finds that the distribution of m^6^A in ccRCC is quite different from normal samples. And it is related to the illness of many cancer-related pathways ranged from HIF-1 signaling pathway, to tight junction and metabolism pathways [102]. Another study also notes the crucial role of m^6^A abnormity in ccRCC, and two underlying mechanisms have been mentioned. First, the decreased expression of METTL3 and METT14 and/or increased expression of ALKBH5 reduce the m^6^A modification of NANOG mRNA and lead to the enhancement of its translation. As we known, NANOG is a crucial transcription factor for the pluripotency of stem cells. Thus, the proliferation of ccRCC stem cells is out of control. Second, they also note a high frequency of VHL gene silencing and the overexpression of hypoxia induced factor-α (HIF-α) in ccRCC. And they come up with the VHL-HIF-METTL3/METTL14 pathway, devoting to the ccRCC stem cells as well [103]. Tang et al. note the overexpression of WTAP in ccRCC, which is connected with the metastasis by enhancing the CDK2 translation [8]. However, Alexander and his coworkers find that AKBH5 and FTO, the two demethylases, are both downregulated in ccRCC, associated with a poor prognosis [104]. Totally speaking, we have known that m^6^A dysregulation is involved in the development and progression of ccRCC, but the specific mechanism is not completely clear and requires further exploration.

#### Prostate cancer

VIRMA and YTHDF2 play vital roles in prostate cancer, the second most common diagnosed cancer in men [105]. According to a recent study, VIRMA is up-regulated in prostate cancer. It can enhance malignant phenotype and the translation of oncogenic long non-coding RNAs (lncRNAs) CCAT1 and CCAT2 in an m^6^A-dependent way [106]. And the level of YTHDF2 is also up-regulated. Knockdown of YTHDF2 inhibits the proliferation of prostate cancer cells, suggesting YTHDF2 has an oncogenic role. In contrast to pancreatic cancer, YTHDF2 enhances cell motility and is associated with tumor metastasis in prostate cancer [107]. This study further indicates that YTHDF2 has a negative correlation with miR-493-3p, providing a potential therapeutic target [107].

#### Bladder cancer (BC)

As the 10th most common diagnosed cancer worldwide, BC causes about 200,000 deaths a year especially in men [108]. The abnormal m^6^A has been proved to be involved in the occurrence and development of BC. A study finds that the expression of METTL3, YTHDF1 and HNRNPC are increased in BC, however, FTO is down-regulated. The expression of VIRMA and ALKBH5 in BC are not different from that of normal tissue [109]. Mechanistically, a recent study notes the overexpression of METTL3 and YTHDF2, and provides a novel underlying mechanism of BC. In their opinion, the up-regulated METTL3 leads to the increased m^6^A methylation of SETD7 and KLF4, two cancer suppressors. And it facilitates the recognition by YTHDF2, which is responsible for degradation [110]. Another study also shows the increased expression of METTL3, YTHDF1 and YTHDF3, and offers a different mechanism. From their points of view, METTL3 interacts with the 3’UTR of ITGA6 mRNA and fulfills the task of methylation. And then the m^6^A-modified ITGA6 is recognized by YTHDF1 and YTHDF3, which promotes its translation. And the ITGA exerts its tumor supporter role in BC [111]. What’s more, a recent study points out that the down-regulation of METTL14 in BC and bladder tumor initiating cells (TICs). The knockdown of METTL14 enhances the ability of TICs in BC, suggesting the suppressive role of METTL14 in BC. In mechanism, METTL14 plays its suppressive role by inducing the m^6^A-modified Notch-1 to inhibit the translation by destabilizing it. The Notch-1 is known as a crucial factor for TICs self-renewal [112].

### Reproductive system

#### Cervical cancer (CC) and endometrial cancer (EC)

Like prostate cancer in men, CC is the second most frequent diagnosed malignancy among women [113]. The therapeutic effect of CC is not satisfactory primarily due to the chemoradiotherapy resistance. And several studies have shown the critical role of m^6^A methylation in CC. It is found that lncRNA GAS5-AS1 can enhance the stability of GAS5, a tumor suppressor, through interacting with ALKBH5. ALKBH5 reduces the degradation of GAS5 by abolishing the recognition by YTHDF2 in an m^6^A-dependent way. However, the level of lncRNA GAS5-AS1 decreases significantly, and instead, the expression of YTHDF2 is up-regulated, which diminishes the role of GAS5 and eventually causes the occurrence of CC [114]. Another study notes the marked elevated level of FTO in CC, and emphasizes its importance in the chemoradiotherapy resistance. FTO inhibits the translation of β-catenin mRNA by decreasing its m^6^A methylation, which leads to the resistance of chemoradiotherapy [115].

EC has a similar incidence to CC, with a high mortality rate [113]. The level of m^6^A methylation is proved to be down-regulated in EC. Liu and his coworkers point out that the decreased expression of METTL3 and/or METTL14 R298P mutation should be responsible for this regulated m^6^A methylation. And the down-regulated m^6^A methylation devotes to the excessive activation of AKT via the following two ways. First, it reduces m^6^A methylation of PHLPP2, a negative AKT regulator, which leads to the decreased recognition by YTHDF1. Usually, YTHDF1 can enhance the stability of m^6^A-modified PHLPP2 mRNA and facilitate its translation. Second, the down-regulated m^6^A -modified mTORC contributes to its decreased degradation in an YTHDF2-dependent way, and finally increases the expression of mTORC, an optimistic AKT regulator. Eventually, the dysregulated m^6^A methylation leads to EC [116].

#### Ovarian cancer (OC)

OC is also one of the cancers that afflicts women, and has connection with abnormal m^6^A methylation. A study notes the positive correlation between the up-regulated METTL3 and the overexpression of RHPN1-AS1 in OC. And it has been proved that METTL3 enhances the expression of RHPN1-AS1 in an m^6^A-dependent way. Furthermore, RHPN1-AS1 can facilitate the proliferation and metastasis of OC cells through playing a role as a ceRNA to sponge miR-596, up-regulating the expression of LEMT1 and leading to the excessive activation of FAK/PI3K/Akt pathway [117].

#### Breast cancer

There is no doubt that the breast cancer exerts its bothersome role to women, with an awfully high incidence and mortality. Like ccRCC, hypoxia is involved in the occurrence of breast cancer by inducing the dysregulation of m^6^A methylation. The up-regulated ALKBH5 caused by hypoxia, decreases the m^6^A methylation of NANOG mRNA and promotes its expression, finally leading to the power of breast cancer stem cells (BCSCs) [118]. Moreover, the decrease of METTL3 in breast cancer has been noted. Mechanistically, METTL3 is inhibited by zinc finger protein 217 (ZFP217), which is also induced by hypoxia. Then the decrease of METTL3 and increase of ALKBH5 together lead to the overexpression of NANOG and KLF4, another BCSCs supporter [119].

On the contrary, three other studies show the up-regulation of m^6^A methylation in breast cancer, and provide different underlying mechanisms. One of them finds the increased METTL3 enhances the expression of hepatitis B X-interacting protein (HBXIP), which facilitates the self-renew of BCSCs. To our surprise, HBXIP can promote the overexpression of METTL3 in turn by inhibiting the antioncogene let-7 g. In this way, the positive feedback of HBXIP/let-7 g/METTL3/HBXIP is formed, which greatly accelerates the progression of breast cancer [120]. Interestingly, another study shows the unusual function of up-regulated m^6^A methylation to switch the role of MAGI3 from antioncogene to an oncogene, which leads to the breast cancer [121]. And the last one claims that the up-regulated METTL3 contributes to breast cancer via targeting Bcl-2 mRNA and enhancing its translation [122].

Recently, a new research indicates the oncogenic role of VIRMA in breast cancer. According to this research, the up-regulated VIRMA targets its downstream mRNAs like cyclin-dependent kinase 1 (CDK1) mRNA and enhances its translation in an m^6^A-dependent way to promote the progression of breast cancer [123]. And the ability of 5′-fluorouracil (5-FU) to reduce the expression of VIRMA and CDK1 has been certified, which offers a possible treatment for breast cancer.

### Circulatory system

#### Hematological malignancies

Acute myeloid leukemia (AML), derived from leukemia stem cells or progenitor cells, causes more than 10,000 deaths a year, whose 5-year survival rates are 26% for adults and 65% for children [124, 125]. The role of FTO in AML has already gained attention, especially in some specific situations like MLL-rearrangement, FLT-ITD3, PML-RARα, NPM1 mutation and t (15,17) [126]. Recently, with the increasing notice of m^6^A, other m^6^A regulators have been gradually found to be related to AML, such as METTL3, METTL14, ALKBH5 and YTHDF2.

The level of METTL3 is elevated in AML. It can interact with the promoter of SP1 mediated by CEBPZ and enhance the function of SP1 to activate oncogene c-MYC [127]. Moreover, METTL3 can also promote the translation of Bcl-2 and PTEN mRNA. These two mechanisms promote the proliferation of leukemia cells and inhibit their differentiation, leading to AML [128]. Both as a writer, the level of METTL14 is also up-regulated like METTL3 to exert its oncogenic role. Mechanistically, METTL14 targets oncogenes MYB and MYC in m^6^A-dependent way and then promotes their translation to make contribution to AML [129].

The expression of FTO is also increased in AML. FTO can inhibit the expression of some tumor suppressors including ankyrin repeat, retinoic acid receptor α (RARα) and SOCS box protein 2 (ASB2) by reducing their m^6^A methylation, which is linked to the development of AML and resistance of all-trans-retinoic acid (ATRA) treatment in M3 (French-American-British type) [126]. In another mechanism, FTO decreases the degradation of oncogenic MYC and CRBPA mRNA in YTHDF2-dependent way by demethylation [130]. Thus, FTO finishes its duty as an oncogenic role in AML. However, both as an eraser, ALKBH5 is controversial in AML. Some studies demonstrate that ALKBH5 is down-regulated in AML and it represents a worse outcome and may correlate with TP53 mutation [131]. On the contrary, two recent studies reveal that ALKBH5 plays an oncogenic role in AML [132, 133]. These two studies also find that ALKBH5 is over-expressed in AML and promotes the self-renewal of leukemia stem/initiating cells (LSCs/LICs). In mechanism, ALKBH5 participates in post-translation regulation of TACC3, a recognized oncogene in various cancers. It is shown that ALKBH5 promotes the expression of TACC3 via enhancing its stability in an m^6^A-dependent way [132]. Moreover, it is proved that KDM4C can facilitate recruitment of MYB and Pol II to regulate the expression of ALKBH5. Besides, ALKBH5 down-regulates the expression of AXL via increasing the level of m^6^A, which conduces to the proliferation of LSCs/LICs [133].

R-2-hydroxyglutarate (R-2HG), a tumor suppressor, can inhibit the expression of FTO, providing us a novel imagination in AML treatment [130]. We can also investigate whether MA2, which have been applied to the treatment of GBC as an FTO inhibitor, could make contribution to AML treatment.

## Conclusions and perspectives

**Fig. 3:**
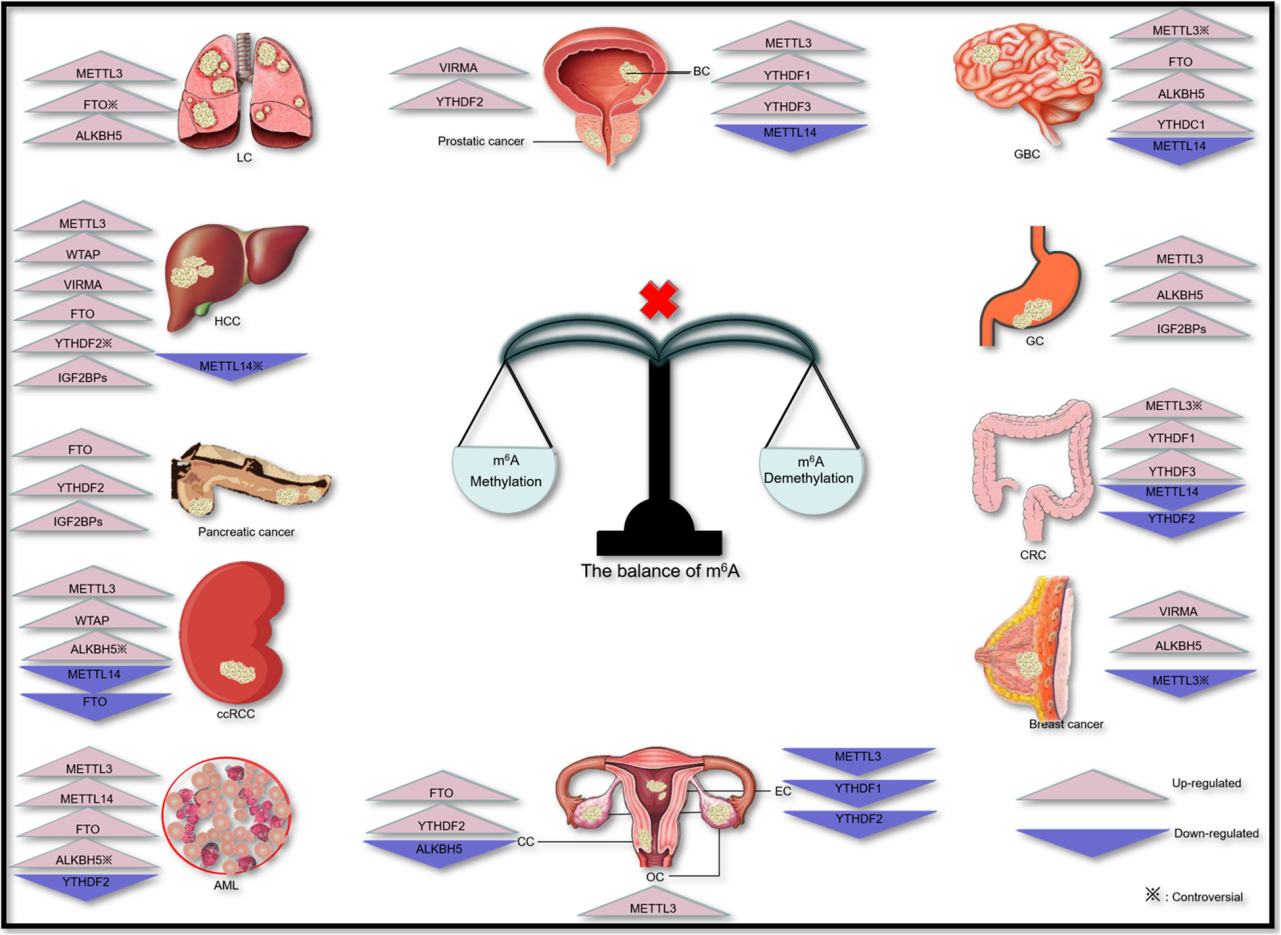
Aberrant expression of m^6^A regulators in different cancers

The role of m^6^A in cancers has been confirmed by many studies, ranging from solid cancers to hematology malignancies (Table 1 and Fig. 3). Therein, the importance of m^6^A in cancers is indisputable. Most abnormal m^6^A regulators play a role in promoting tumorigenesis. Primarily, they exert their oncogenic roles by inhibiting the expression of antioncogenes or enhancing the expression of oncogenes to promote the proliferation, metastasis and invasion of cancer stem cells, such as the function of METTL3 in GBCs and ALKBH5 to BCSCs [58, 59, 118, 119]. Nevertheless, the role of m^6^A in cancers is not limited to the promotion of tumorigenesis. It is more like a double-edged sword, and sometimes also functions as tumor suppressor, like METTL14 in ccRCC and YTHDF2 in EC [103, 116].

**Table 1:** The role of m^6^A regulators in different cancers

m^**6**^A regulators	Cancer type	Role in cancer	Genes involved	Mechanism	Reference
**Writers**					
METTL3	GBC^a^	Oncogene	SOX2	Enhances the stemness of GSCs by facilitating expression of SOX2	[58]
			SRSFs	Enhances the stemness of GSCs by increasing the NMD of SRSFs in YTHDC1-dependent way	[59]
		Antioncogene	ADAM19	Inhibits the stemness of GSCs by promoting the expression of ADAM19	[60, 61]
	LC	Oncogene	EGFR TAZ MK2 DNMT3	Enhances the stemness of LC stem cells by promoting expression of EGFR, TAZ, MK2 and DNMT3	[53]
			VASH1	Promotes brain metastasis of LC by inhibiting the expression of VASH1 via up-regulated miR-143-3p	[70]
	HCC	Oncogene	SOCS2	Increases the decay of tumor suppressor SOCS2 via YTHDF2 in m^6^A-dependent way	[76]
			Snail	Promotes the progress of EMT by regulating the expression of Snail in YTHDC1-dependent way	[77]
			HDGF	Up-regulates the level of LINC00958 and promotes the expression of HDGF by inhibiting miR-3619-5p	[78]
	GC	Oncogene	MYC	Enhances the expression of oncogene MYC	[86]
			Bcl2	Inhibits the apoptosis of GC cells by up-regulating the expression of Bcl2 via competitive inhibition with Bax	[10]
			SEC62	Promotes the expression of SEC62 with the help of IGF2BP1	[88]
			HDFG	Promotes the expression of HDFG with the help of IGF2BP3	[89]
	CRC^a^	Oncogene	SOX2	Enhances the proliferation of CRC cells by facilitating expression of SOX2 in a m^6^A-IGF2BP2-dependent mechanism	[92, 93]
		Antioncogene	?	Inhibits the proliferation, metastasis in CRC by suppressing the p38/ERK signaling pathways	[94]
	ccRCC	Antioncogene	NANOG	Inhibits the stemness of ccRCC cells by reducing the expression of NANOG	[103]
			VHL	Inhibits the stemness of ccRCC cells by up-regulating the expression of VHL and reducing the level of HIF-α	[103]
	BC	Oncogene	SETD7 KLF4	Facilitates the degradation of tumor suppressor m^6^A-modified SETD7 mRNA and m^6^A-modified KLF4 mRNA in YTHDF2-dependent way	[110]
			ITGA6	Promotes the expression of oncogene ITGA6 in YTHDF1 and YTHDF3 dependent way	[111]
	EC	Antioncogene	PHLPP2 mTORC	Promotes the excessive activation of AKT by reducing expression of PHLPP2 and increasing expression of mTORC	[116]
	OC	Oncogene	RHPN1-AS1	Promotes the expression of RHPN1-AS1 and activates FAK/PI3K/Akt pathway	[117]
	Breast cancer^a^	Antioncogene	NANOG	Inhibits the stemness of BCSCs by reducing the translation of NANOG	[119]
			KLF4	Inhibits the stemness of BCSCs by reducing the translation of KLF4	[119]
		Oncogene	HBXIP	Enhances the stemness of BCSCs by increasing the expression of HBXIP	[120]
			MAGI3	switches the role of MAGI3 from antioncogene to an oncogene in m^6^A-dependent way	[121]
			Bcl2	Promotes the expression of Bcl2	[122]
	AML	Oncogene	SP1 c-MYC	Promotes the expression of SP1 and enhance its function to activate oncogene c-MYC	[127]
		Oncogene	Bcl2 PTEN	Promotes the proliferation of leukemia cells and inhibits their differentiation by enhancing expression of Bcl2, PTEN	[128]
METTL14	GBC	Antioncogene	ADAM19	Inhibits the stemness of GSCs by promoting the expression of ADAM19	[60, 61]
	HCC^a^	Antioncogene	miR126	Inhibits the metastasis and relapse of HCC by promoting the mature of tumor suppressor miR126 with the aid of DGCR8	[83]
	CRC	Antioncogene		Inhibits the proliferation and metastasis of CRC by up-regulating the m^6^A level of lncRNA XIST and facilitates its degradation	[95]
	ccRCC	Antioncogene	NANOG	Inhibits the stemness of ccRCC cells by reducing the expression of NANOG	[103]
			VHL	Inhibits the stemness of ccRCC cells by up-regulating the expression of VHL and reducing the level of HIF-α	[103]
	BC	Antioncogene	Notch-1	Inhibits the self-renewal of TICs by reducing the expression of Notch-1	[112]
	AML	Oncogene	MYB MYC	Promotes the expression of MYB and MYC	[129]
WTAP	HCC	Oncogene	ETS1	Inhibits the expression of HCC suppressor ETS1 with the help of HuR	[79]
	ccRCC	Oncogene	CDK2	Promotes the metastasis by increasing the expression of CDK2	[8]
VIRMA	HCC	Oncogene	ID2	Promotes the migration by decreasing the expression of ID2	[80]
			GATA3	Promotes decay of GATA3 by inducing the separation of HuR	[81]
	Prostate cancer	Oncogene	CCAT1, CCAT2	Enhances malignant phenotype and the translation of lncRNAs CCAT1, CCAT2	[106]
	Breast cancer	Oncogene	CDK1	Enhances the expression of CDK1	[123]
**Erasers**					
FTO	GBC	Oncogene	ADAM19	Enhances the stemness of GSCs by promoting the expression of ADAM19	[58]
	LC^a^	Oncogene	LAMC2 TSP1 VGF ITGα11 PCSK9	Enhances the stemness of lung cancer stem cells by promoting expression of LAMC2, TSP1, VGF, ITGα and PCSK9	[67]
	HCC	Oncogene	PK2	Promotes the expression of PK2 by demethylation	[82]
	Pancreatic cancer	Oncogene	MYC,bHLH-TF	Promotes the proliferation of pancreatic cancer cells by increasing expression of MYC, bHLH-TF	[98]
	ccRCC	Antioncogene	?	?	[104]
	CC	Oncogene	?	Enhances the resistance to chemoradiotherapy by inhibiting the translation of β-catenin mRNA	[115]
	AML	Oncogene	RARα ASB2	Inhibits differentiation of AML cells and promotes resistant to ATRA by enhancing the expression of RARα and ASB2	[126]
		Oncogene	MYC CRBPA	Reduces the degradation of MYC and CRBPA in YTHDF2-dependent way	[130]
ALKBH5	GBC	Oncogene	ADAM19 FOXM1	Enhances the stemness of GSCs by promoting the expression of ADAM19 and FOXM1	[33, 58, 62]
	LC	Oncogene	FOXM1	Enhances the stemness of GSCs by promoting the expression of FOXM1	[33, 62, 69]
	GC	Oncogene	NEAT1	Promotes the metastasis by increasing the expression of NEAT1	[90]
	ccRCC^a^	Oncogene	NANOG	Promotes the stemness of ccRCC cells by increasing the expression of NANOG	[103]
			VHL	Promotes the stemness of ccRCC cells by silencing VHL and increases the level of HIF-α	[103]
		Antioncogene	?	?	[104]
	CC	Antioncogene	GAS5	Inhibits the decay of tumor suppressor GAS5 by interacting with lncRNA GAS5-AS1 in YTHDF2-dependent way	[114]
	Breast cancer	Oncogene	NANOG	Enhances the stemness of BCSCs by promoting expression of NANOG	[118, 119]
			KLF4	Enhances the stemness of BCSCs by promoting the expression of KLF4	[119]
	AML^a^	Oncogene	TACC3 AXL	Promotes self-renewal of LSCs/LICs via ALKBH5/m^6^A/TACC3 axis and KDM4C/ALKBH5/AXL axis	[132, 133]
		Antioncogene	TP53	Inhibits the mutation of TP53	[131]
**Readers**					
YTHDF1	CRC	Oncogene	?	Promotes the proliferation of CRC cells by activating the Wnt/beta-catenin pathway	[96]
	BC	Oncogene	ITGA6	Promotes the translation of ITGA6 by enhancing its stability	[111]
	EC	Antioncogene	PHLPP2	Keeps the balance of AKT pathway by promoting the expression of PHLPP2	[116]
YTHDF2	HCC^a^	Oncogene	SOCS2	Facilitates the degradation of tumor suppressor m^6^A-modified SOCS2 mRNA and down-regulate its expression	[76]
		Antioncogene	IL11 SERPINE2	Improves the microenvironment by accelerating the degradation of IL11, SERPINE2	[84]
	CRC	Antioncogene	XIST	Inhibits the proliferation and metastasis of CRC by facilitating the degradation of m^6^A-modified lncRNA XIST	[95]
	Pancreatic cancer	Oncogene	?	Promotes the progression of EMT by regulating the YAP signaling pathway and inhibits the metastasis of pancreatic cancer	[100]
	Prostate cancer	Oncogene	?	Promotes the proliferation of prostate cancer cells and accelerates metastasis by?	[107]
	CC	Oncogene	GAS5	Promotes the degradation of tumor suppressor GAS5	[114]
	EC	Antioncogene	mTORC	Keeps the balance of AKT pathway by promoting the decay of mTORC	[116]
	AML	Antioncogene	MYC CRBPA	Accelerates the degradation of MYC CRBPA	[130]
YTHDF3	BC	Oncogene	ITGA6	Promotes the translation of ITGA6 by enhancing its stability	[111]
	CRC	Oncogene		Facilitates the progression of CRC by the negative function loop of lncRNA GAS5-YAP-YTHDF3 axis	[97]
YTHDC1	GBC	Oncogene	SRSFs	Enhances the stemness of GSCs by promoting the decay of m6A-modified SRSFs mRNA	[59]
		Oncogene	Snail	Promotes the progression of EMT by regulating the expression of Snail	[77]
IGF2BPs	HCC	Oncogene	MYC	Promotes the expression of oncogene MYC by enhcancing its stability	[51]
	GC	Oncogene	SEC62	Promotes the translation of m6A-modified SEC62 mRNA by enhancing its stability	[88]
			HDFG	Promotes the translation of m6A-modified HDGF mRNA by enhancing its stability	[89]
	Pancreatic cancer	Oncogene	DANCR	Enhances the stemness of pancreatic cancer cells by up-regulating the expression of DANCR	[99]
METTL3	LC	Oncogene	BRD4	Promotes the expression of oncogene BRD4 by interacting with eIF3h	[71]

^a^There is still some controversy

We note that m^6^A regulators can play the same role in different cancers by the same or different way. Also, it can play two opposite roles in different cancer. For example, METTL3 is a tumor promoter in OC by enhancing the expression of RHPN1-AS1, while it is a suppressor in EC via over activating the AKT signaling pathway [116, 117]. There is some controversy like the role of METTL3 in GBC, CRC and breast cancer, partially due to the differences in primary tumor specimens and heterogeneity of cell line epigenetics.

It’s interesting to find that both as classic writer, METTL3 acts as a tumor promoter most of the time, while METTL14 acted as a tumor suppressor most of the time. This may be explained by their different downstream targets and the different functions of readers. Inexplicably, METTL3 and METTL14 sometimes regulate in opposite directions. For instance, METTL3 is up-regulated in GBC, while METTL14 is down-regulated according to some studies [58–61]. However, as mentioned in the part of “The process and molecular functions of m^6^A RNA methylation”, METTL3 is combined with METTL4 at 1:1 to form a heterodimer and exert their methylated function with the help of other writers [18]. Whatever their downstream genes are, it is confusing that how they cooperate with each other when levels are so incoordinate, which deserves further study.

With the rapid development of inspection technology, more and more m^6^A regulators are gradually recognized, including the discovery of new members and the excavation of new functions of old members. The roles of some new members in cancers like CBLL1 have not been studied and mechanisms of some m^6^A regulators in cancers are not very clear and they need more researchers to focus on. And the discovery of METTL3 as a reader provides us more possibilities to explore new functions of other old members [71].

In general, the role of m^6^A has attracted much attention. Comfortingly, m^6^A lives up to our expectations and provides various possibilities to further study. As a matter of fact, m^6^A already has clinical implications not only as a diagnostic and prognostic biomarker but also as a therapeutic target. However, what we have known is only the tip of the iceberg. With the help of developing technologies, the role of m^6^A in cancers will be more thoroughly studied.

## Data Availability

Not applicable.

## Abbreviations

m^6^A: N6-methyladenosine
UTRs: Untranslated regions
METTL3: Methyltransferase-like 3
SAM: S-adenosylmethionine
METTL14: Methyltransferase-like 14
WTAP: Wilms tumor 1-associated protein
VIRMA: Virilizer like m^6^A methyltransferase associated protein
RBM15: RNA binding motif protein 15
ZC3H13: Zinc finger CCCH-type containing 13
CBL11: Cb1 proto-oncogene like 1
METTL16: Methyltransferase-like 16
FTO: Fat mass and obesity-associated protein
ALKBH5: AlkB homologue 5
ALKBH3: AlkB homologue 3
YTH: YT521-B homology
YTHDF: YTH domain family protein
YTHDC: YTH domain containing protein
HNRNP: Heterogeneous nuclear ribonucleoproteins
IGF2BPs: Insulin-like growth factor 2 mRNA-binding proteins
eIF3: Eukaryotic initiation factor 3
LRPPRC: Leucine rich pentatricopeptide repeat containing
FMRP: Fragile X mental retardation protein
GBM: Glioblastoma
GSCs: Glioma stem cells
HuR: Human antigen R
LC: Lung cancer
LUAD: Lung adenocarcinoma
HCC: Hepatocellular carcinoma
GC: Gastric cancer
CRC: Colorectal cancer
ccRCC: Clear cell renal cell carcinoma
BC: Bladder cancer
CC: Cervical cancer
EC: Endometrial cancer
OC: Ovarian cancer
AML: Acute myeloid leukemia
LSCs/LICs: Leukemia stem/initiating cells
